# Influence of Microbial Treatment on the Preparation of Porous Biochar with Stepped-Up Performance and Its Application in Organic Pollutants Control

**DOI:** 10.3390/ijms232214082

**Published:** 2022-11-15

**Authors:** Yingjie Su, Keyu Xie, Jiaohui Xiao, Siji Chen

**Affiliations:** 1 College of Life Sciences, Jilin Agricultural University, Changchun 130118, China; 2 Key Laboratory of Straw Comprehensive Utilization and Black Soil Conservation, Ministry of Education, Jilin Agricultural University, Changchun 130118, China

**Keywords:** microbial treatment, biochar, organic pollutants, enhanced performance, application

## Abstract

In this study, *Irisensata Thunb* grass (ITG) was used as a biomass carbon resource to prepare biochars for the first time. After microbial treatment, the obtained microbial-treated ITG (MITG) was activated by using a mixed base as an activator for preparation of biochar (MITGB). The specific surface area and total pore volume of MITGB were 3036.4 m^2^/g and 1.5252 cm^3^/g, which were higher than those of biochar prepared without microbial treatment (ITGB, 2930.0 m^2^/g and 1.5062 cm^3^/g). Besides, the physicochemical properties of MITGB and ITGB were also quite different including micro morphology, surface chemistry, functional groups, etc. In the experiment of removing organic pollutants with synthetic dye RhB and antibiotic TH as the models, MITGB showed excellent treatment ability. The maximum adsorption capacities of MITGB for RhB and TH were 1354.2 and 1462.6 mg/g, which were higher than most of the biochars. In addition, after five cycles of recycling, the adsorption capacities of the organic pollutant models can still be maintained at more than 80%, which showed high stability. This work verified the feasibility of microbial treatment to further improve the performance of biochar and provided a new idea and direction for exploring other biochars.

## 1. Introduction

Environmental problems have become a topic of concern all over the world, especially water pollution, which is the top priority [1,2]. In today’s era, our efforts to develop social productivity, and improve living standards, but also caused serious damage to the environment, especially with the acceleration of the process of industrialization, a large number of industrial wastewater is discharged into the environment, which contains a large number of toxic organic pollutants, such as synthetic dyes and antibiotics [3,4]. Among many organic pollutants, there are up to 300 organic pollutants that pose a threat to human beings [5]. In addition, data surveys show that the pollution rate of sediments in lakes and reservoirs in China has exceeded 80% [6]. These organic pollutants not only have high color and difficulty in natural degradation but also have strong teratogenicity, carcinogenicity, and other problems, which seriously threaten people’s health [7,8,9]. How to reduce pollution, protect the ecological balance and solve environmental problems has attracted the attention of scholars at home and abroad. 

Therefore, developing a simple and effective method to control water pollution is a top priority for human society. In recent years, with the development of science and technology, a series of advanced treatment technologies have been derived, such as biodegradation [10], advanced oxidation technology [11], and membrane separation [12]. Among them, the adsorption method using biochar as an adsorbent has become one of the most popular technologies for water pollution treatment due to its low cost, no by-products, mild conditions, and easy operation [13,14]. Biochar is a kind of solid material generated from biomass under anaerobic or anoxic conditions. It has a heterogeneous and highly aromatic structure. Its huge specific surface area and highly developed pore structure lay an unparalleled foundation for its application in the field of adsorbents [15,16].

In general, the properties of biochar are mostly determined by its natural structure and preparation technology. The unique natural structure and more advanced preparation technology often maximize the potential of exploiting biochar properties [17,18]. Grass has attracted more and more attention from biochar researchers due to its unique structure (long lignofibrous structure, abundant natural pores, and abundant functional groups, etc.). In the past few decades, many kinds of grass-based biochar have been developed with different forage materials, and all of them have quite good performance [19,20,21]. *Irisensata Thunb* grass (ITG), is a perennial herb widely planted in China, which has rich and tough leaves in grayish-green strips. It is not only one of the important packaging materials of traditional food (Zongzi) in Northeast China, but also a biomass resource with high yield and large reserves. Over the past five years, our research team has been exploring the utilization of biomass resources, developing carbon sources for the preparation of new biochar (such as cow dung biochar [13], lantern fruit shell biochar [22], fungal mycelium biochar [14,23,24], etc.), and advanced technologies to improve biochar performance (such as lignocellulose separation [25,26], mixed alkali activation [27,28], etc.). However, to date, there have been very few studies on biochar preparation from this abundant grass resource, *ITG*, which gives us a great incentive to exploit this valuable biomass resource.

The present study developed a novel strategy for preparing biochar with improved performance. In short, the *Irisensata Thunb* grass was pretreated by microbial fungi to change the natural structure of the biomass. Based on the previous foundation of our team [26], we found that the treatment of biomass by microorganisms mainly depends on whether the main component of biomass is lignocellulose or not, and the effect of microbial treatment depends on the content of lignocellulosic components. Then, the microbial-treated ITG-based biochar material (MITGB) was prepared by carbonization and mixed base activation. Subsequently the synthetic dye (Rhodamine B, RhB) and antibiotics (Tetracycline hydrochloride, TH) were selected as model organic pollutants to explore the governance performance of MITGB to organic pollutants in a water environment (including the effects of pH, adsorption kinetics, adsorption isotherm, and adsorption thermodynamics, etc.), also has a contrast test different preparation methods (the ITG based biochar material without microbiological treatment, ITGB) on the effects of performance. Finally, the adsorption mechanism was studied and discussed. The aim of this study is to explore the impact of microbial treatment on biochar material preparation, so as to provide a rapid and effective way to further improve biochar performance and better cope with organic pollutants control.

## 2. Results and Discussion

### 2.1. Preparation of MITGB

**Scheme 1:**
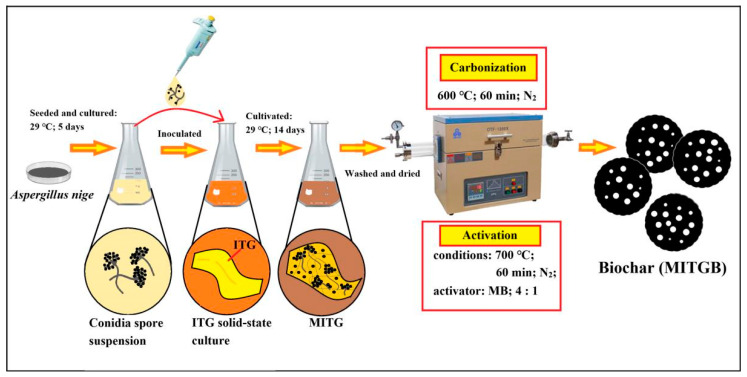
Schematic plot of the preparation of MITGB.

The preparation process of MITGB is shown in Scheme 1 and can be divided into two stages including microbial treatment of ITG and preparation of biochar. Microbial treatment of ITG was performed by solid-state fermentation in a flask [29]. Similar to most agricultural wastes, such as corn stover, ITG is also a major component of lignocellulose [25,26], and *Aspergillus Niger* (*A. niger*), which can degrade lignocellulosic components of ITG [26], is used as a tool strain for microbial treatment in this step. The *A. niger* was seeded into a flask containing PD liquid medium and cultured at 29 °C and 150 RPM for 5 days to produce a conidia spore suspension containing a large number of fungal spores and mycelium. Then, the conidia spore suspension was inoculated into the solid-state fermentation medium which contained raw material of ITG and cultivated for 14 days to degrade the lignocellulosic components and destruct the natural compact lignocellulosic structure of ITG [30]. After that, the MITG was carbonized and activated in a horizontal tubular furnace to prepare biochar materials.

### 2.2. Results of Characterization

**Figure 1:**
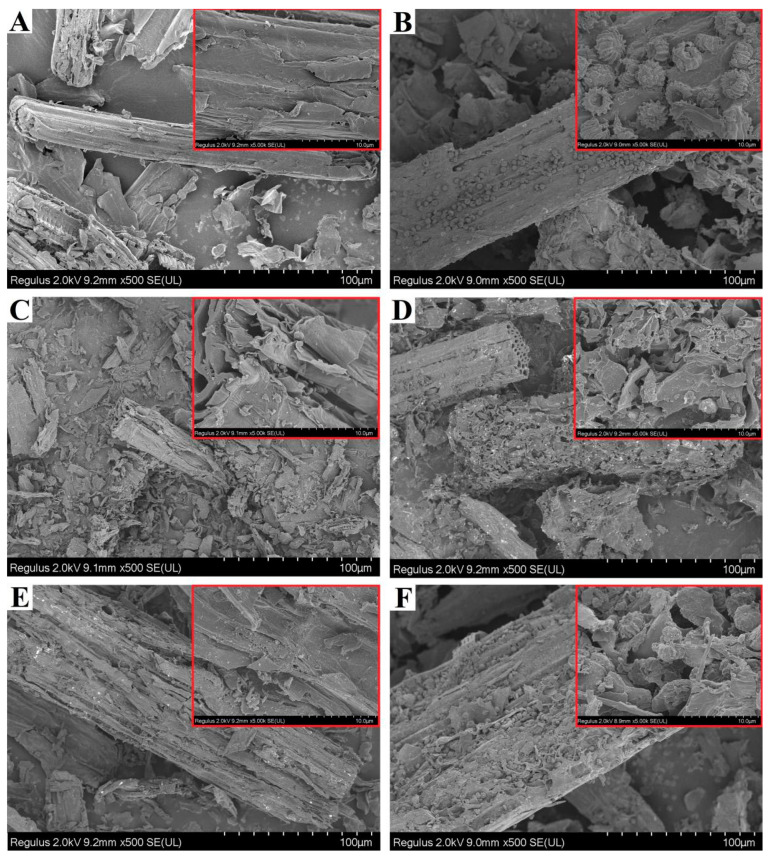
SEM of (**A**) ITG, (**B**) MITG, (**C**) CITG, (**D**) CMITG, (**E**) ITGB, and (**F**) MITGB.

The microscopic morphology of the sample was observed by SEM and shown in Figure 1. The surface of the original ITG is relatively smooth and the dense wood fiber texture structure can be seen. After microbial treatment, the surface began to appear rough and uneven, and it can be speculated that *A. niger* does play a role in the process of ITG treatment. After the high-temperature carbonization process, the surface of CITG and CMITG were become rough and occurred some uneven folds and fragmentation. In addition, there are some heterogeneous pore structures, especially CMITG is more obvious. Subsequently, CITG and CMITG undergo intense high-temperature activation reaction with the activator MB, which further cracks the wood structure still visible after carbonization, intensified the degree of surface fragmentation, and finally formed biochars with porous structure (ITGB and MITGB).

**Figure 2:**
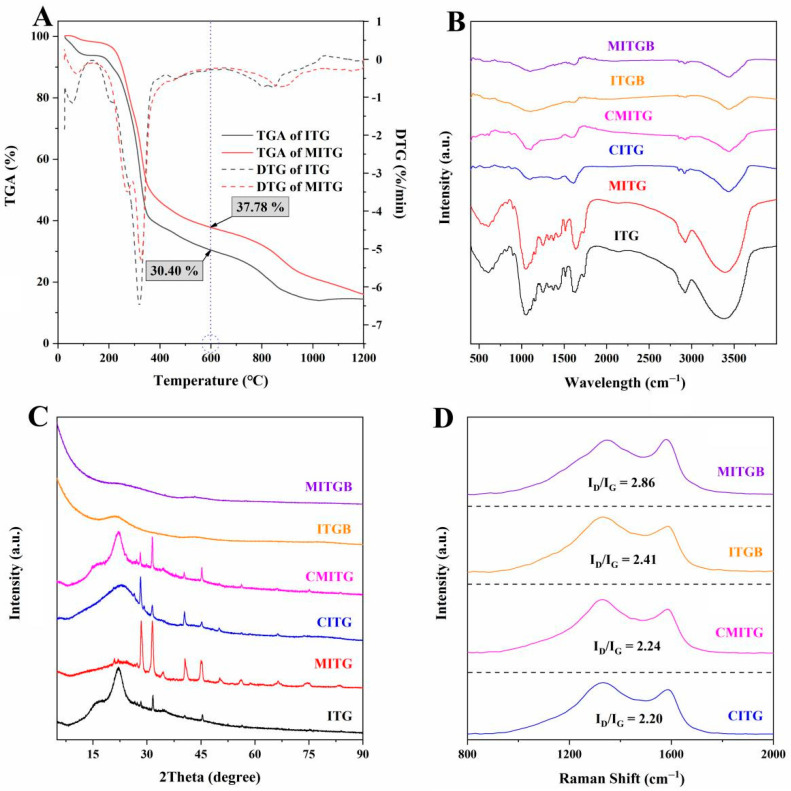
(**A**) TGA curves of ITG and MITG. (**B**) FT-IR spectra and (**C**) XRD pattern of samples. (**D**) Raman spectra of CITG, CMITG, ITGB, and MITGB.

The influence of temperature on ITG and MITG was obtained by TGA tests under nitrogen protection as shown in Figure 2A. The TGA curves correspond to the different stages of biochars formation, which indicates that there are three main stages of weight loss in the range from room temperature to 1200 °C. The first stage occurred at room temperature to 200 °C, which was caused by evaporation and loss of residual water from physical surfaces and internal pores [22,23]. Like many other biomasses, the main oxygenated components of ITG and MITG are cellulose, hemicellulose, lignin, protein, and fat, which could be broken into gas and tar during the high-temperature pyrolysis process [22,23,24]. The removal of these pyrolytic materials results in the weight loss of the second stage. As for the third stage (from 600 to 1200 °C), especially when temperatures exceed 800 °C, the weight loss was mainly due to the cleavage of some minerals [31,32]. Compared with ITG, the pyrolysis behavior of MITG was more obvious, which indicated that the mineral composition of MITG was higher than that of ITG. It can be speculated that there were two reasons, on the one hand, the residual *A. niger* spores after microbiological treatment, and on the other hand, the residue of inorganic salt culture medium added in the process of microbial treatment. In order to obtain carbonized samples more stably, the carbonization temperature was chosen to be 600 °C, in the meantime, the carbonization yields of CITG and CMITG were 30.40 and 37.78%, respectively.

The functional groups of the sample were analyzed by FT-IR spectra test as shown in Figure 2B. The broad bond at 3384–3436 cm^−1^ represents stretching vibrations of hydroxyl functional groups (O-H). The bond at 2919–2928 cm^−1^ represents symmetrical and asymmetrical stretching vibrations of -CH, -CH^2^, and -CH^3^ groups [22,23,24,25,26,27,28]. The bond at 1729–1731 cm^−1^ represents the stretching vibration of C=O. The bond at 1601–1635 cm^−1^ represents the axial deformation of the carbonyl group (C=O). The bond at 1374–1375 cm^−1^ represents the C-H symmetric bending vibration of the methyl group [14,22]. The bonds at 1251–1252 cm^−1^ and 1419–1421 cm^−1^ represent the deformation vibration of methylene. The bonds around 1047–1159 cm^−1^ represent the tensile vibrations of C-O from alcohols, phenols, acids, or esters [23,24]. Interestingly, the new bond at 1517 cm^−1^ from MITG represents the stretching vibration of a mononuclear aromatic hydrocarbon [33]. It can be inferred that the degradation of macromolecular organic materials does occur after the microbial treatment by *A. niger*, and thus the stretching vibration of the mononuclear aromatic hydrocarbon and olefin occurs simultaneously.

The crystal structure of the sample was tested by XRD as shown in Figure 2C. The peaks at 17 and 23° represent the cellulose from lignocellulose of raw ITG [25,26]. The irregular peaks represent minerals that can be interpreted as inorganic salt culture medium added during microbial treatment (e.g., MgSO_4_, CaCl_2_, FeSO_4_, MnSO_4_, etc.). After carbonization, these peaks become more sharp and distinct [13]. After further treatment (activation), these peaks are basically weakened or even disappeared, which may be because of the pickling and washing links in the preparation process [13,25]. It is worth noting that both ITGB and MITGB exhibited diffraction peaks within the range from 10° to 30°, assigned to the plane of graphite structure (002), which may be indicating that biochars had a classic local-order structure of carbon materials [34,35]. 

The presence of defects in the carbon was tested by Raman spectra as shown in Figure 2D. Two typical peaks were obtained from the results including the D-bond with amorphous carbon around 1350 cm^−1^ and the G-band with graphitic carbon around 1550 cm^−1^ [22,23,24,25,26,27,28]. To measure the degree of defect and disorder in carbons, the intensity ratio of the D-band and G-band (I_D_/I_G_) was used as an important index. The I_D_/I_G_ values of CITG and CMITG were 2.20 and 2.24. After activation, the I_D_/I_G_ values of CITG and CMITG were 2.41 and 2.86, which indicated that more amorphous carbon structures were generated in the biochars.

**Figure 3:**
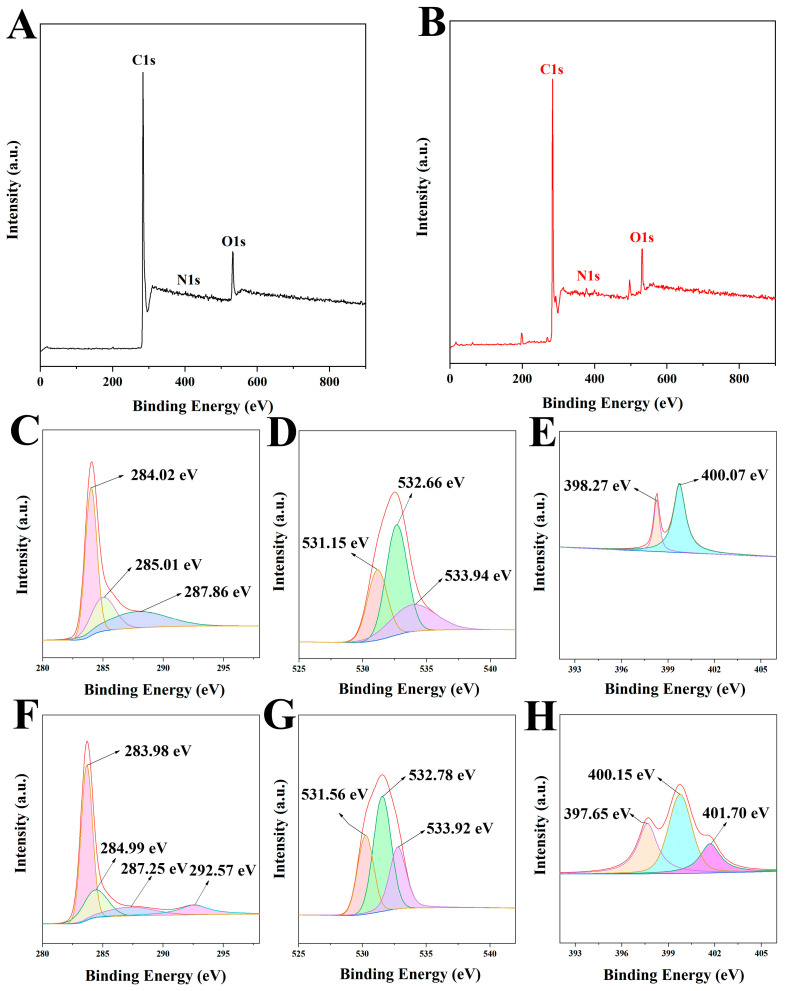
XPS spectra of (**A**) ITGB and (**B**) MITGB. The (**C**) C1s, (**D**) O1s, and (**E**) N1s of ITGB. The (**F**) C1s, (**G**) O1s, and (**H**) N1s of MITGB.

The surface chemical and electronic state of the sample was tested by XPS spectroscopy as shown in Figure 3. Both ITGB and MITGB contain mainly C, O, and N elements, further fitting these elements can obtain different functional keys. The high-resolution C1s spectrum of ITGB showed three classical peaks, which were obtained at 284.02, 285.01, and 287.86 eV corresponding to C-C, C-O, and C=O, respectively [13,14]. The high-resolution C1s spectrum of MITGB showed four peaks, which were obtained at 283.98, 284.99, 287.25, and 292.57 eV corresponding to C-C, C-O, C=O, and C from the transition of π-π, respectively [13,14]. The high-resolution O1s spectrum of ITGB and MITGB both have three peaks at 531.15–531.56, 532.66–532.78, and 533.92–533.94 eV corresponding to C=O, C-O, and -OH, respectively [13,14]. The high-resolution N1s spectrum of ITGB and MITGB both have peaks at 398.27–399.65 eV and 400.07–400.15 eV corresponding to pyridinic-N and pyrrolic-N [13]. Besides, MITGB has a special peak at 401.70 eV corresponding to graphite-N [36], which indicated that the microbial treatment indeed changed partially of the physico-chemical properties of biomass (from ITGB to MITGB).

**Figure 4:**
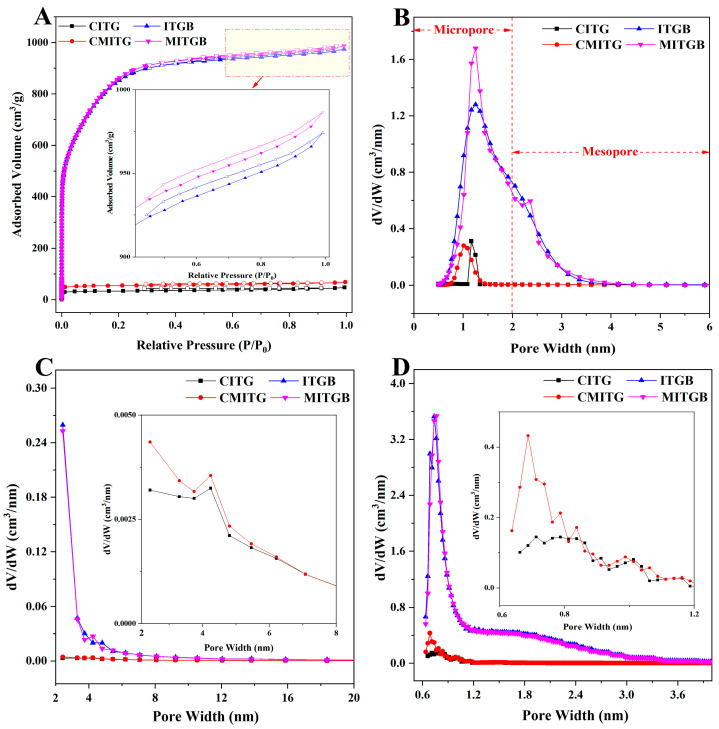
The (**A**) N_2_ adsorption-desorption isotherms and pore size distribution curves of CITG, CMITG, ITGB, and MITGB based on (**B**) NLDFT method, (**C**) BJH method, and (**D**) H-K method.

The specific surface area and porosity of the sample were tested by N_2_ adsorption-desorption isotherms as shown in Figure 4 and Table 1. The specific surface area and the total pore volume of CITG were 129.9 m^2^/g and 0.0729 cm^3^/g, meanwhile, the specific surface area and the total pore volume of MITG were 213.1 m^2^/g and 0.1048 cm^3^/g. It can be boldly speculated that the structure of the carbonized precursor MITG of CMITG has changed significantly after microbial treatment. Although the specific surface area of CMITG increased by nearly 83.2 m^2^/g and the total pore volume increased by about 43.76% compared with CITG, these data are not enough to support CMITG as porous biochar for adsorption research. Therefore, further activation treatment is needed to greatly improve its properties and enhance its application performance.

The pore-forming effect of activators commonly used in biochar preparation (such as NaOH and KOH) is beyond doubt, therefore, MB was used as an activator for biochar preparation in order to comprehensively improve pore structure (both micropore and mesopore structures) [25,27,28]. While MB was used as an activator and ionized to M^+^ (K^+^ and Na^+^) and OH^−^ during the pyrolysis process. These metal ions and hydroxide would react with the carbonized samples and etch them to form carbonate. After washing with dilute acid and deionized water, the microporous and mesoporous structures were finally formed in porous carbon [27,28]. As can be seen from the results that ITGB and MITGB both exhibited typical type IV isotherms with H3 hysteresis loop [13], which indicated that mesoporous structures existed in the biochar prepared. The micropore volumes were 1.4777 cm^3^/g for ITGB and 1.4607 cm^3^/g for MITGB, respectively, accounting for 98.1% and 95.8% of the total pore volume, which indicated that a large number of micropore structures were also present. The specific surface areas and the total pore volumes were 2930.0 m^2^/g and 1.5062 cm^3^/g for ITGB and 3036.4 m^2^/g and 1.5252 cm^3^/g for MITGB, respectively. This indicates that the physico-chemical properties of biomass after microbial treatment are relatively stable and reliable (CITG compared with MITG and ITGB compared with MITGB), and also indicates the feasibility of this microbial treatment-based technology for the preparation of biochar with higher performance. In addition, the pore size distribution results of biochars based on either model algorithm (NLDFT, BJH, and HK) show that both ITGB and MITGB have microporous and mesoporous structures. The above data show that ITGB and MITGB are porous biochar after activation, which can be used as adsorbents to treat organic pollutants in water.

### 2.3. Results of Adsorption Experiments

**Figure 5:**
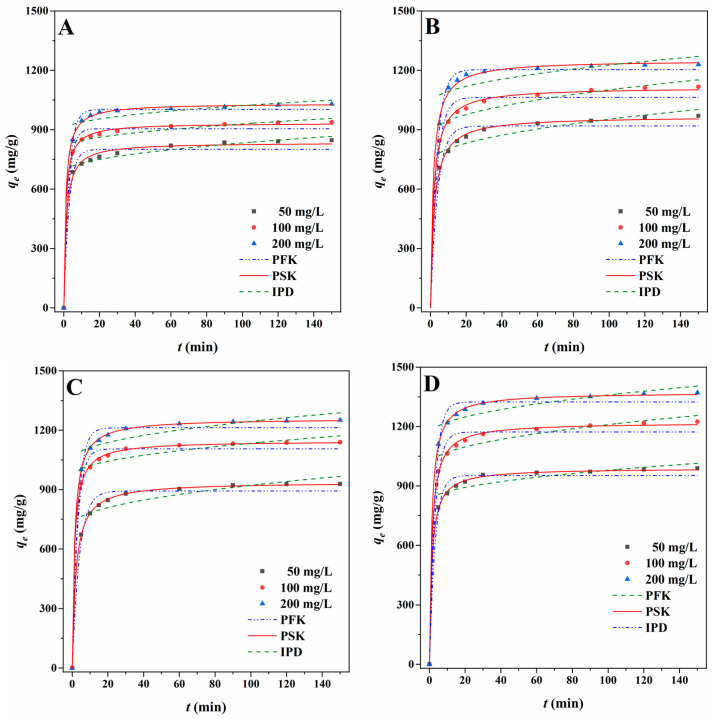
PFK, PSK, and IPD plots of RhB and TH for ITGB (**A**,**B**) and MITGB (**C**,**D**) at 303 K.

Adsorption kinetics describes the change of adsorbent with contact time [37], thus, the contact time on adsorption capacities of ITGB and MITGB for RhB and TH were performed with different initial concentrations of solutions at 303 K as shown in Figure 5. It can be easily found that the trend of all adsorption processes is the same for both ITGB and MITGB, namely rapid adsorption in the first 10 min, followed by a gradual equilibrium adsorption in 30 min, and further extension of contact time does not lead to a more significant increase in adsorption capacity. In addition, the adsorption capacity also increased significantly with the increase in the initial concentration of the solution, indicating that the high concentration of the solution was effective for the improvement of the adsorption capacity to a certain extent.

Besides, to study the control mechanism of chemical reactions in the adsorption process, three common adsorption kinetics models were used to analysis to experimental data as shown in Table 2. Lagergren’s PFK model was used to investigate the adsorption process, the correlation coefficients *R*^2^ of ITGB were range from 0.9784 to 0.9962 for RhB and range of 0.9852–0.9907 for TH, meanwhile, the *q_e.cat_* were both slightly lower than *q_e_* obtained from experiments, which indicated that the PFK model may not play an essential role in the adsorption process. While Weber–Morris’s IPD model, the correlation coefficients *R*^2^ of ITGB ranged from 0.6291 to 0.9083 for RhB and range of 0.7101–0.7368 for TH, showed that and the adsorption processes were not controlled by particle diffusion. While Ho–McKay’s PSK model was used to fit the data, the correlation coefficients *R*^2^ of ITGB ranged from 0.9954 to 0.9996 for RhB and range of 0.9997–0.9999 for TH, at the same time, the *q_e.cat_* (844.5, 937.2, and 1035.8 mg/g for RhB, 938.8, 1145.7, and 1259.2 mg/g for TH) were both suitable with that of obtained from experiments (841.0, 936.6, and 1030.3 mg/g for RhB, 928.0, 1137.9, and 1246.2 mg/g for TH), which showed that the applicability of PSK in the adsorption process. As for MITGB, the correlation coefficients *R*^2^ of the PFK, PSK, and IPD models were 0.9350–0.9957, 0.9982–0.9986, and 0.5491–0.8103 for RhB, 0.9865–0.9911, 0.9990–0.9998, and 0.7352–0.8005 for TH, therefore, the PSK was the most suitable model to describe the adsorption process. According to the results, a conclusion can be inferred that the adsorption processes of ITGB and MITGB for RhB and TH may be chemical reactions [38], and the adsorption behaviors between adsorbent and adsorbate through transfer, exchange, or sharing to form chemisorption bonds may control the adsorption rate [13].

**Figure 6:**
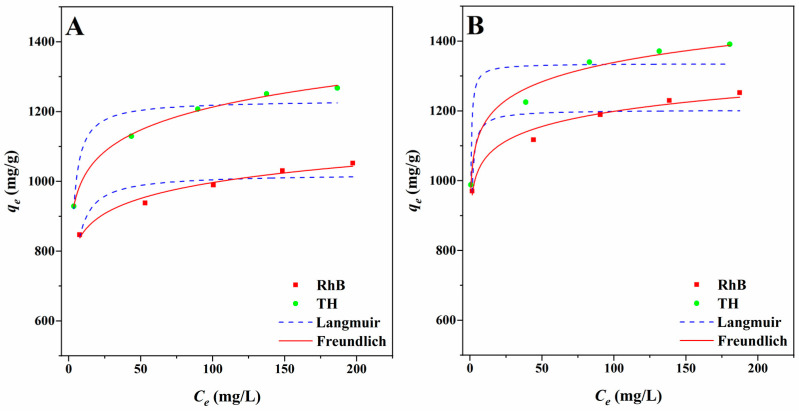
Langmuir and Freundlich isotherms of RhB and TH for (**A**) ITGB and (**B**) MITGB at 303 K.

The effect of concentration on the adsorption capacity of an adsorbent always is studied by investigating the adsorption isotherms [39,40]. Different initial concentrations of solutions were used to explore the behaviors between the adsorbent (ITGB and MITGB) and the adsorbate (RhB and TH) during the processes at 303 K as shown in Figure 6. As can be seen from the results that the adsorption capacities of samples were increased with the increase in the initial concentrations solutions. Langmuir isotherm and Freundlich isotherm models were used to investigate the experimental data as shown in Table 3. 

Langmuir isotherm model was often used to describe the homogeneous molecule adsorption process, the correlation coefficients *R*^2^ were 0.81 for RhB and 0.80 for TH of ITGB, and 0.82 for RhB and 0.86 for TH of MITGB. Freundlich isotherm model was often used to study the heterogeneous multilayer adsorption process, the correlation coefficients *R*^2^ were 0.98 and 0.99 for ITGB to RhB and TH, and 0.97 and 0.98 for MITGB to RhB and TH. By comparing the correlation coefficients *R*^2^ of Langmuir with that of Freundlich, it can be inferred that the adsorption processes of both RhB and TH were not uniform single-layer adsorption processes, but non-uniform multi-layer adsorption processes [41]. Furthermore, the intensity factors *n_F_* of ITGB and MITGB were 14.28 and 18.87 for RhB, 12.50 and 14.46 for TH, which also indicated that the process should be multilayer adsorption on heterogeneous surfaces [13].

**Figure 7:**
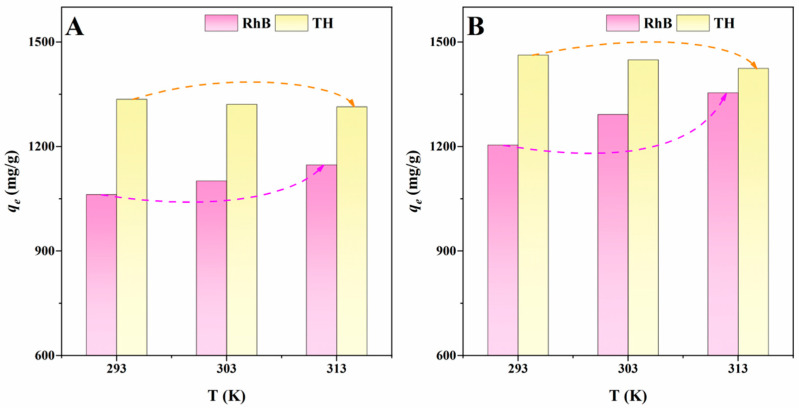
Adsorption thermodynamic of (**A**) ITGB and (**B**) MITGB.

Temperature is one of the most important factors affecting the adsorption process [42], the influence of different temperatures (293, 303, and 313 K) on organic pollutants adsorption by biochar is shown in Figure 7. At 293 K, the adsorption capacities of ITGB and MITGB for RhB were 1062.1 and 1204.4 mg/g, respectively, and 1335.8 and 1462.6 mg/g for TH, respectively. With the increase in temperature to 303 K, the adsorption capacities of RhB increased, on the contrary, the adsorption capacities of TH decreased. When the temperature further increases to 313 K, the trend is still the same. Obviously, the rise of temperature promoted the adsorption process of RhB and inhibited the adsorption of TH, that is, the influence of a high-temperature environment on the adsorption of organic pollutants by biochars (ITGB and MITGB) was different.

In order to further explore the influence of temperature, the experimental data were analyzed by the thermodynamic model and the parameters as shown in Table 4. All Δ*G* values were negative, indicating that the adsorption occurs spontaneously [43] regardless of ITGB or MITGB and whether RhB or TH is adsorbed. The thermodynamic enthalpy Δ*H* of adsorption were 3.41 and 5.32 kJ/mol for RhB, and −0.75 and −1.23 kJ/mol for TH, which further confirmed the endothermic property of the RhB adsorption process and the exothermic property of TH adsorption process [44]. In addition, the positive values of thermodynamic Δ*S* (20.94 and 28.69 Jmol^−1^ K^−1^ for RhB, 9.00 and 8.64 J mol^−1^ K^−1^ for TH) indicated that the randomness and chaos of the interface between porous biochars and solutions increased with the increase in temperature [45].

**Figure 8:**
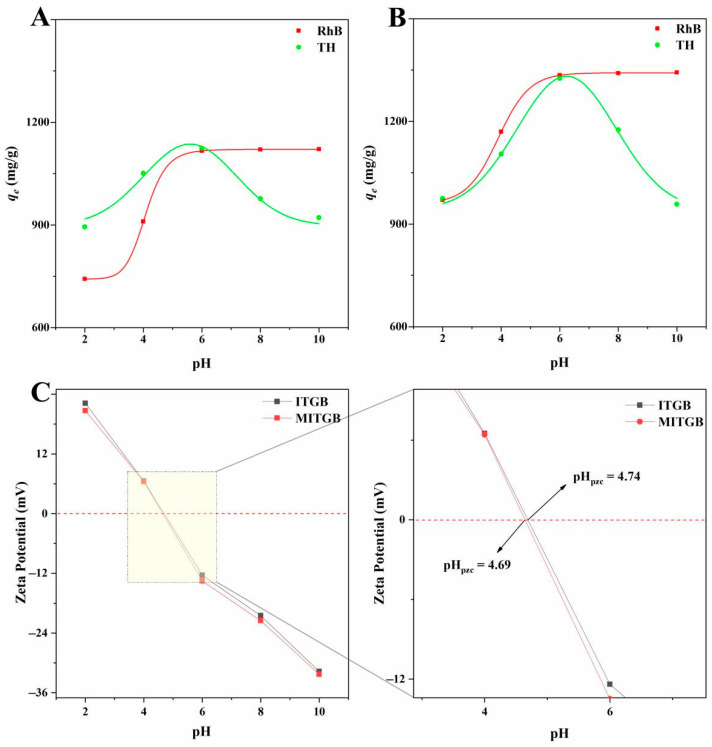
Effect of pH on the adsorption capacities of (**A**) ITGB and (**B**) MITGB. (**C**) Zeta potential of ITGB and MITGB.

The pH of the solution environment is an important factor affecting the adsorption process, that is, by affecting the surface properties of the adsorbent and the chemical properties of the adsorbed pollutant molecules to promote or inhibit its adsorption capacity [22,23]. The effect of pH in the range of 2–10 on the removal ability of organic pollutants was studied as shown in Figure 8. It can be easily seen that with the increasing of the initial pH of the solutions, the RhB removal ability of both ITGB and MITGB increased first and then leveled off, while for TH adsorption the trend increased first and then decreased.

RhB is a cationic dye, and the presence of a large amount of H^+^ in the solution will compete with the dye for adsorption sites on the surface of biochars at lower pH [13,14]. On the other hand, when the pH was less than pH_pzc_ of biochars (4.74 for ITGB and 469 for MITGB), the surfaces of biochars were positively charged, which will also repel cationic dyes [14]. With the increase in pH, especially while the pH was greater than pH_pzc_ of biochars, the surface of biochars started to be filled with a negative charge, and electrostatic attraction promoted the adsorption capacities of cationic dye RhB. In addition, it was found that the improvement of RhB adsorption capacity at higher pH was not endless. While the pH value was higher than 6, the increase in the pH value did not continue to significantly improve the abilities of biochars to remove RhB, which indicated that the electrostatic attraction can promote the adsorption of RhB, but it was not the only force affecting the adsorption process [27]. As for the adsorption of TH by biochars, the obvious influence of pH may be that it not only affected the surface electrochemical properties of biochars, but also had a great influence on the molecular properties of TH [13,14,24]. Generally speaking, TH exists in different forms under different pH environments. When the pH value was lower than 3.30, TH was mainly TH^+^, and the Zeta potential of biochars was positive, at this time, the mutually repulsive electrostatic attraction inhibited the adsorption process. When pH was greater than 3.30 but less than 7.68, TH existed in the form of TH^+−^ or TH^0^. While pH was greater than 7.68 but less than 9.68, TH existed in the form of TH^−^. However, when pH was greater than 9.68, TH was mostly TH^2−^, which is the most unfavorable for adsorption with biochars (ITGB and MITGB) [13,14,24].

### 2.4. Results of Cycle Tests and Comparison with Other Biochars

**Figure 9:**
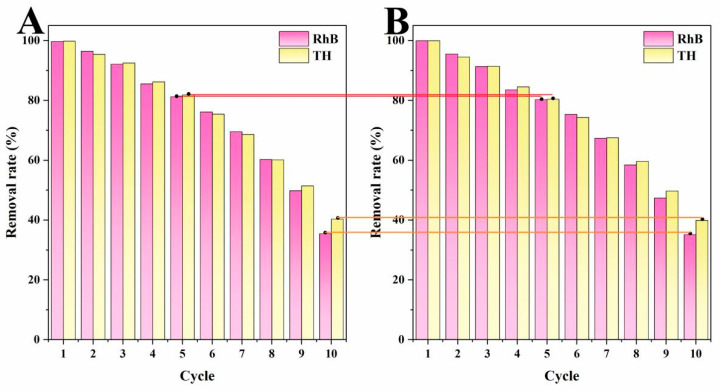
Cycling stability of (**A**) ITGB and (**B**) MITGB.

The recyclability of adsorbents was one of the important parameters to evaluate the practical performance of biochar adsorbents [22,23,24,25,26,27,28]. Biochars were investigated under the five-cycle experiments as shown in Figure 9. The removal rates of RhB and TH by ITGB and MITGB decreased with the increasing cycle times. After five cycles, the removal rates of RhB and TH by ITGB were still 81.2 and 81.8%, and the removal rates of MITGB were still kept at more than 80%. After ten cycles, the removal rates of RhB by ITGB and MITGB were 35.4 and 35.1%, and the removal rates of TH were kept at 40.3 and 39.9%. It can be explained by the fact that on the one hand, with the processing of cycle experiments, adsorbed organic pollutants form by-products on the surface of biochars. On the other hand, with the increasing re-carbonization times, the texture and structure of the regenerated biochars become more fragile, which may cause partial pore structure breakage, and then reduce the specific surface area, thus further affecting the adsorption performances [13]. Although the removal rate of MITGB was slightly lower than that of ITGB, it can still be concluded that ITGB and MITGB have good stability and recycling ability for the removal of organic pollutants (RhB and TH).

The adsorption capacity of the adsorbent was also one key parameter to evaluate the practical performance, thus, the adsorption capacities of ITGB and MITGB were compared with other biochar adsorbents with highly BET-specific surface areas as shown in Table 5. It can be easily seen from the experimental results that the BET-specific surface area and adsorption capacity of ITGB and MITGB were not low, which fully indicated that the biochars prepared had great potential and application prospects in organic pollutants treatment.

**Table 5:** Comparison of ITGB and MITGB to RhB and TH with other biochars.

Adsorbents	S_BET_ (m^2^/g)	*q_e_* for RhB (mg/g)	*q_e_* for TH (mg/g)	References
ITGB	2930.0	1147.3	1335.8	This work
MITGB	3036.4	1354.2	1462.6	This work
Bagasse pith biochar	522.7	263.9	−	[46]
Rice husk biochar	892.0	518.1	−	[47]
Edible fungus substrate biochar	2767.3	1497.0	−	[28]
Cow dung biochar	4081.1	1241.0	1105.0	[13]
*Aspergillus niger*/starch biochar	3050.0	1188.2	1386.0	[14]
*Eucommia ulmoides* lignin−based biochar	2008.0	−	1163.0	[48]
Fungal mycelium−agar biochar	4343.3	−	1441.3	[24]
Cellulose biochar	2583.0	−	1854.2	[26]

### 2.5. Possible Mechanisms Discussion

**Figure 10:**
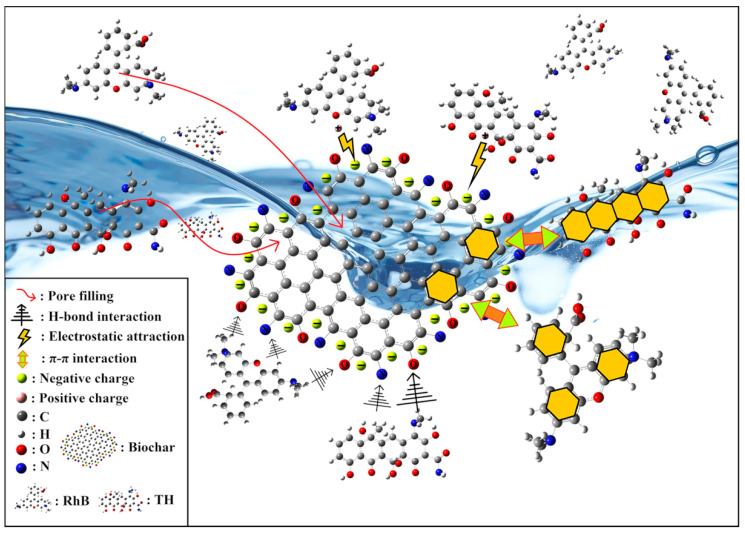
Probable mechanisms of MITGB for removal of organic pollutants.

In this study, the large specific surface area and high total pore volume (3036.4 m^2^/g and 1.5252 cm^3^/g) of MITGB provided a large number of adsorption sites for organic pollutants (RhB and TH). The average pore size of MITGB was 1.98 nm, which was larger than the molecular size of RhB (1.2 nm) and TH (1.3 nm), therefore, it was speculated that pore filling may play an important role in the adsorption process. The analysis results of FT−IR spectra and XPS spectra showed that there were a large number of unsaturated functional groups containing carbon, oxygen, and nitrogen on the surface of MITGB, and these functional bonds were likely to form hydrogen bonds between biochar with pollutant molecules, thus improving the removal ability. In addition, aromatic rings (such as alkenes and mononuclear aromatic hydrocarbons) exist in MITGB, which may also have π−π interaction with aromatic rings in pollutants to enhance their adsorption capacity. Finally, in the appropriate pH environment, the charged organic pollutants can form a strong electrostatic attraction with MITGB, which further promotes the adsorption process. Thus, it can be speculated that MITGB exhibits strong removal ability of organic pollutants in water under the joint action of pore filling, π−π interaction, H-bond interaction, and electrostatic attraction (Figure 10).

## 3. Materials and Methods

### 3.1. Materials and Reagents

*Irisensata Thunb* grass (ITG) was obtained from the experimental area of Jilin Agricultural University (Changchun, China) in 2022, washed with deionized water, dried at 80 °C for 12 h, and crushed. *Aspergillus niger* (*A. niger*) was supported by the Key Laboratory of Straw Comprehensive Utilization and Black Soil Conservation, Ministry of Education.

D-glucose monohydrate and agar were provided by Sinopharm Chemical Reagents Co., Ltd. (Shanghai, China). Potatoes were bought from a local supermarket. (NH_4_)_2_SO_4_, KH_2_PO_4_, MgSO_4_, CaCl_2_, NaCl, FeSO_4_, MnSO_4_, ZnCl_2_, NaOH, HCl, and KOH were purchased from Beijing Chemical Works (Beijing, China) and used without further purification. Mixed base (MB) was prepared by using equal masses of NaOH and KOH. 

Rhodamine B (RhB, CAS: 81−88−9) and Tetracycline hydrochloride (TH, CAS: 64−75−5) were supplied by Aladdin Chemical (Shanghai) Co. Ltd. (Shanghai, China) and the structural formulas of RhB and TH are shown as Figure S1 (Supplementary Material).

### 3.2. Microbial Treatment of ITG

In a typical process, 10 g of ITG and 30 mL of fermentation nutrient solution ((NH_4_)_2_SO_4_: 2.0 g/L, KH_2_PO_4_: 2.0 g/L, MgSO_4_: 0.3 g/L, CaCl_2_: 0.3 g/L, NaCl: 0.5 g/L, FeSO_4_: 0.005 g/L, MnSO_4_: 0.016 g/L, and ZnCl_2_: 0.017 g/L) were thoroughly mixed, sterilized at 115 °C for 30 min, and cooled down to room temperature. The microorganisms were cultured in a potato dextrose medium (PD, potato extract: 200 g/L, D-glucose monohydrate: 20.0 g/L) at 29 °C for 5 days. Then, a 2% conidia spore suspension (*v*/*w*) of microorganisms (*A. niger*) was inoculated into the prepared ITG solid-state culture. The system was sealed and cultivated at 29 °C for 14 days and the solid residue was collected and washed with 0.1% NaOH solution (*v*/*w*) to remove the *A. niger*. After that, the precipitate was washed with deionized water until a neutral pH and dried at 80 °C for 12 h. 

### 3.3. Preparation of MITGB

The obtained microbial-treated ITG (MITG) was carbonized at 600 °C for 60 min with a heating rate of 10 °C/min under the protection of a nitrogen atmosphere. After the carbonized MITG (CMITG) cooled to room temperature, the CMITG was mixed with MB activator at a ratio of 4:1 and heated at 700 °C for 60 min. Then the activated mixture was washed with HCl and deionized water until reaching a pH value of about 7. Afterwards, the biochar material (MITGB) was dried and kept in a desiccator prior to subsequent experiments. The abbreviations CITG and ITGB represent the carbonized ITG and ITG-based biochar materials without microbiological treatment. The characterization methods are shown in Supplementary Material.

### 3.4. Adsorption Performances

In a batch adsorption experiment, 0.10 g/L of ITGB or MITGB was added to a flask containing organic pollutant solutions (RhB or TH). The flask was placed in a constant temperature shaker at 150.0 RPM in the dark. After the adsorption process reached equilibrium, the suspension was centrifuged and diluted with the supernatant. The concentration of the solution was determined by an Agilent Cary−300 UV−vis spectrophotometer. The adsorption capacities of samples were calculated by Equation (1):

(1) $$q_{e}=\frac{(C_{0}-C_{e})\times V}{M}$$

where *q_e_* (mg/g) is the adsorption capacity of the sample, *C_e_* and *C*_0_ (mg/L) denote the equilibrium and initial concentrations of the solution. *M* (g) and *V* (L) denote the mass of the samples and the volume of the solutions, respectively.

The organic pollutant solutions were prepared at different concentrations (50, 100, and 200 mg/L). 0.05 g/L of ITGB or MITGB were dispersed into flasks containing RhB or TH solutions and shaken with 150 RPM in the dark at 303 K. Then, the concentrations of the solutions were determined at preset time intervals. The pseudo-first-order kinetic (PFK, Equation (2)), the pseudo-second-order kinetic (PSK, Equation (3)), and the Intra-particle diffusion model (IPD, Equation (4)) were used to analyze the adsorption kinetic data, shown as follow:

(2) $$\ln(q_{e}-q_{t})=\ln q_{e}-k_{1}t$$

(3) $$\frac{t}{q_{t}}=\frac{1}{k_{2}q_{t}^{2}}+\frac{t}{q_{e}}$$

(4) $$q_{t}=k_{3}t^{0.5}+C$$

where *q_t_* and *C* are the adsorption capacity of the sample at different time points *t* and the thickness of the boundary layer. *k*_1_, *k*_2_, and *k*_3_ denote the PFK, PSK, and IPD adsorption kinetic rate constants, respectively.

The organic pollutant solutions at different initial concentrations (50, 100, 150, 200, and 250 mg/L) were prepared and used to test the adsorption isotherm at 303 K. After adsorption saturation, the absorbances of the solutions were measured by UV−vis spectrophotometer. The adsorption isotherm data were investigated by using the Langmuir isotherm model (Equation (5)) and Freundlich isotherm model (Equation (6)), shown as follows:

(5) $$\frac{C_{e}}{q_{e}}=\frac{C_{e}}{q_{m}}+\frac{1}{q_{m}K_{L}}$$

(6) $$\ln q_{m}=\frac{1}{n}\ln C_{e}+\ln K_{F}$$

where *q_m_* (mg/g) is the maximum adsorption capacity of the sample calculated by the adsorption isotherm model, *K_L_* and *K_F_* denote the Langmuir and Freundlich adsorption isotherm constants.

The effect of temperatures (293, 303, and 313 K) on the adsorption capacity of the adsorbates was investigated at an initial concentration of 100 mg/L with ITGB or MITGB of 0.05 g/L. The thermodynamic parameters were analyzed to describe the effect of temperatures on the adsorption process. The calculation equations were shown as follows:

(7) $$\ln\left(K_{T}\right)=-\frac{\Delta H}{RT}+\frac{\Delta S}{R}$$

(8) $$K_{T}=\frac{q_{e}}{C_{e}}$$

(9) ΔG=ΔH−TΔS

where Δ*S*, Δ*G*, Δ*H*, and *R* represent the thermodynamic parameters standard entropy, standard free Gibbs energy, standard enthalpy, and the gas constant (8.314 J/K·mol), respectively.

The variation of the adsorption capacity of the samples with pH (2, 4, 6, 8, and 10) was also investigated. The solutions were adjusted to different pH values by HCl and NaOH.

### 3.5. Cycling Stability Studies

In each cycle, 1.0 g/L of ITGB or MITGB was placed into a flask that contaminant the organic pollutant solutions with a concentration of 100 mg/L. After the adsorption experiment finished, the recycled ITGB or MITGB was washed with water and carbonized for 60 min at 600 °C under the protection of a nitrogen atmosphere. The re-carbonized samples were re−used as fresh adsorbents in the next cycle.

## 4. Conclusions

In this study, ITG was used as a new biomass, after microbial (*A. niger*) treatment, MB was used as an activator for carbonization−activation to prepare biochar. The specific surface area and total pore volume of MITGB were 3036.4 m^2^/g and 1.5252 cm^3^/g, respectively, which were higher than those of ITGB (2930.0 m^2^/g and 1.5062 cm^3^/g). In the experiment of removing organic pollutants with synthetic dye RhB and antibiotic TH as the organic pollutant models, it showed excellent treatment ability. In addition, after 5 cycles of regeneration, the adsorption capacity can still remain above 80%. Finally, the mechanisms that may affect the adsorption process are discussed, mainly pore filling, π−π interaction, H-bond interaction, and electrostatic attraction. This work not only prepared a performance-enhanced biochar (MITGB), which can better treat organic pollutants, but also verified the feasibility of microbial treatment to further improve the performance of biochar and provided a new idea and direction for further exploring other biomass. In the future, we will explore the application of MITGB in more fields, such as the adsorption of smaller impurities such as 1,4−dioxane, etc.

## Figures and Tables

**Table 1:** The data of N_2_ adsorption-desorption for CITG, CMITG, ITGB, and MITGB.

Samples	S_BET_ (m^2^ g^−1^)	V_micro_ (cm^3^ g^−1^)	P_m_ (nm)	V_total_ (cm^3^ g^−1^)
CITG	129.9	0.0575	2.24	0.0729
CMITG	213.1	0.0924	1.97	0.1048
ITGB	2930.0	1.4777	2.08	1.5062
MITGB	3036.4	1.4607	1.98	1.5252

S_BET_ (m^2^ g^−1^), V_micro_ (cm^3^ g^−1^), P_m_ (nm), and V_total_ (cm^3^ g^−1^) represent the BET specific surface area, the volume of micropores, the mean pore size, and the total pore volume.

**Table 2:** Fitting parameters of adsorption kinetic models for RhB and TH at 303 K.

Adsorbates	Adsorbents	Models	Parameters	*C*_0_ (mg L^−1^)		
				50	100	200
RhB	ITGB		*q_e_* (mg/g)	841.0	936.6	1030.3
		PFK	*k*_1_ (min^−1^)	0.0108	0.0110	0.0104
			*q_e.cat_* (mg/g)	801.3	905.4	1003.0
			*R* ^2^	0.9784	0.9889	0.9962
		PSK	*k*_2_ (g mg^−1^ min^−1^)	0.0001	0.0001	0.0001
			*q_e.cat_* (mg/g)	844.5	937.2	1035.8
			*R* ^2^	0.9954	0.9991	0.9996
		IPD	*k*_3_ (mg g^−1^ min^−0.5^)	14.94	12.23	12.21
			*C*	684.1	807.5	900.2
			*R* ^2^	0.9083	0.8114	0.6291
	MITGB		*q_e_* (mg/g)	962.8	1111.8	1227.3
		PFK	*k*_1_ (min^−1^)	0.0064	0.0059	0.0075
			*q_e.cat_* (mg/g)	919.0	1025.5	1203.9
			*R* ^2^	0.9783	0.9350	0.9957
		PSK	*k*_2_ (g mg^−1^ min^−1^)	0.0005	0.0005	0.0005
			*q_e.cat_* (mg/g)	967.5	1114.6	1250.7
			*R* ^2^	0.9984	0.9986	0.9982
		IPD	*k*_3_ (mg g^−1^ min^−0.5^)	21.86	22.57	19.44
			*C*	734.9	876.1	1032.7
			*R* ^2^	0.8099	0.8103	0.5491
TH	ITGB		*q_e_* (mg/g)	928.0	1137.9	1246.2
		PFK	*k*_1_ (min^−1^)	0.0046	0.0098	0.0089
			*q_e.cat_* (mg/g)	893.3	1105.8	1212.8
			*R* ^2^	0.9852	0.9905	0.9907
		PSK	*k*_2_ (g mg^−1^ min^−1^)	0.0005	0.0007	0.0006
			*q_e.cat_* (mg/g)	938.8	1145.7	1259.2
			*R* ^2^	0.9998	0.9997	0.9999
		IPD	*k*_3_ (mg g^−1^ min^−0.5^)	20.26	16.49	19.31
			*C*	719.3	969.8	1051.9
			*R* ^2^	0.7368	0.7208	0.7101
	MITGB		*q_e_* (mg/g)	982.7	1218.2	1365.1
		PFK	*k*_1_ (min^−1^)	0.0089	0.0090	0.0076
			*q_e.cat_* (mg/g)	952.4	1172.6	1324.3
			*R* ^2^	0.9885	0.9865	0.9911
		PSK	*k*_2_ (g mg^−1^ min^−1^)	0.0007	0.0006	0.0006
			*q_e.cat_* (mg/g)	989.7	1220.2	1372.2
			*R* ^2^	0.9994	0.9990	0.9998
		IPD	*k*_3_ (mg g^−1^ min^−0.5^)	15.64	20.43	20.06
			*C*	822.9	1005.8	1158.9
			*R* ^2^	0.7352	0.8005	0.7364

**Table 3:** Fitting parameters of adsorption isotherm models for RhB and TH at 303 K.

Adsorbates	Adsorbents	Types	Parameters	
RhB	ITGB	Langmuir	*q_m_* (mg/g)	1021.5
			*K_L_* (L/mg)	0.5985
			*R* ^2^	0.81
		Freundlich	*K_F_* (mg g^−1^(L mg^−1^)^1/n^)	730.5
			*n_F_*	14.28
			*R* ^2^	0.98
	MITGB	Langmuir	*q_m_* (mg/g)	1202.8
			*K_L_* (L/mg)	2.7126
			*R* ^2^	0.82
		Freundlich	*K_F_* (mg g^−1^(L mg^−1^)^1/n^)	938.7
			*n_F_*	18.87
			*R* ^2^	0.97
TH	ITGB	Langmuir	*q_m_* (mg/g)	1231.4
			*K_L_* (L/mg)	0.8304
			*R* ^2^	0.80
		Freundlich	*K_F_* (mg g^−1^(L mg^−1^)^1/n^)	837.5
			*n_F_*	12.50
			*R* ^2^	0.99
	MITGB	Langmuir	*q_m_* (mg/g)	1335.7
			*K_L_* (L/mg)	4.8893
			*R* ^2^	0.86
		Freundlich	*K_F_* (mg g^−1^(L mg^−1^)^1/n^)	1012.0
			*n_F_*	16.46
			*R* ^2^	0.98

**Table 4:** Fitting adsorption thermodynamic parameters for RhB and TH.

Adsorbents	Adsorbates	T (K)	Δ*G* (kJ/mol)	Δ*H* (kJ/mol)	Δ*S* (J mol^−1^ K^−1^)
ITGB	RhB	293	−2.73	3.41	20.94
		303	−2.92		
		313	−3.14		
	TH	293	−3.38	−0.75	9.00
		303	−3.46		
		313	−3.56		
MITGB	RhB	293	−3.08	5.32	28.69
		303	−3.40		
		313	−3.66		
	TH	293	−3.65	−1.23	8.24
		303	−3.75		
		313	−3.81		

## Data Availability

The study did not report any data.

## Supplementary Materials

The supporting information can be downloaded at: https://www.mdpi.com/article/10.3390/ijms232214082/s1.
